# Investigating the effects of the aging brain on real tool use performance—an fMRI study

**DOI:** 10.3389/fnagi.2023.1238731

**Published:** 2023-08-22

**Authors:** Clara Seifert, Jingkang Zhao, Marie-Luise Brandi, Thabea Kampe, Joachim Hermsdörfer, Afra Wohlschläger

**Affiliations:** ^1^Chair of Human Movement Science, Department of Sport and Health Sciences, Technical University of Munich, Munich, Germany; ^2^Department of Electrical and Computer Engineering, Technical University of Munich, Munich, Germany; ^3^Department of Neuroradiology, TUM-Neuroimaging Center, Technical University of Munich, Munich, Germany; ^4^Graduate School of Systemic Neurosciences, Ludwig-Maximilians-University Munich, Munich, Germany

**Keywords:** tool use, brain representation, apraxia, healthy aging, fMRI

## Abstract

**Introduction:**

Healthy aging affects several domains of cognitive and motor performance and is further associated with multiple structural and functional neural reorganization patterns. However, gap of knowledge exists, referring to the impact of these age-related alterations on the neural basis of tool use–an important, complex action involved in everyday life throughout the entire lifespan. The current fMRI study aims to investigate age-related changes of neural correlates involved in planning and executing a complex object manipulation task, further providing a better understanding of impaired tool use performance in apraxia patients.

**Methods:**

A balanced number of sixteen older and younger healthy adults repeatedly manipulated everyday tools in an event-related Go-No-Go fMRI paradigm.

**Results:**

Our data indicates that the left-lateralized network, including widely distributed frontal, temporal, parietal and occipital regions, involved in tool use performance is not subjected to age-related functional reorganization processes. However, age-related changes regarding the applied strategical procedure can be detected, indicating stronger investment into the planning, preparatory phase of such an action in older participants.

## Introduction

The use of objects as tools represents a fundamental aspect of human’s everyday life. It enables us to achieve goals, to interact with our environment and to accomplish everyday demands. Several diseases predominantly occurring in the older population impact brain regions specifically dedicated to tool use, described as the tool use network. Among these are stroke (Goldenberg and Spatt, 2009), corticobasal degeneration (Gross and Grossman, 2008), dementia (Lesourd et al., 2013) or Parkinson’s disease (Bohlhalter and Osiurak, 2013). The characteristic inability of using tools in the context of these diseases has been termed apraxia. Since they typically occur at higher age, it is of particular interest to specifically study the neural basis of tool use performance in older subjects.

### fMRI in tool use

Aiming at the investigation of brain activation patterns underlying tool use, fMRI data can help to reveal the localization and degree of involved related neural processes. However, the application of fMRI poses several challenges with regards to MRI compatibility of usable tools, or movement artifacts elicited by handling of objects within the MRI. Therefore, only a few studies exist investigating brain activation patterns in response to real 3D tools, utilized in everyday life (Brandi et al., 2014; Styrkowiec et al., 2019; Knights et al., 2021). Brandi et al. (2014) focused on the investigation of brain activation during action planning and execution by the implementation of a tool carousel, containing everyday tools and their respective counterparts. Knights et al. (2021) examined how typical grasping of an actual object induces brain activity in hand- and tool-selective areas. Another study published previously (Styrkowiec et al., 2019) focused on the involvement of brain regions in functional grasping of everyday objects. Besides these particular studies, multiple research findings, addressing tool-related mental processing, consistently reveal a network of left-sided brain areas including several occipital, temporal, parietal and frontal regions being involved in tool use performance (Johnson-Frey et al., 2005; Lewis, 2006; Hermsdörfer et al., 2007; Vingerhoets et al., 2009; Valyear et al., 2012; Gallivan et al., 2013; Brandi et al., 2014; Styrkowiec et al., 2019; Knights et al., 2021; Michalowski et al., 2022). The relevance of the left hemisphere for tool-related actions is additionally confirmed by several lesion studies, including apraxia patients (Goldenberg and Spatt, 2009; Goldenberg, 2013; Salazar-López et al., 2016). Besides investigating the brain lesion’s location, particular attention was paid to the patients’ handedness, language areas and how both relate to apraxic symptoms manifestation. As tool-related actions contain a complex interplay of cognitive and sensorimotor abilities (Johnson-Frey et al., 2005), it is relevant to investigate the influence of an individuals’ handedness on tool use performance. Evidence indicates, that apraxia is dissociated from the hemispheric dominance for handedness (Goldenberg, 2013) and core regions of the praxis representation network, such as the left inferior parietal lobe, play a crucial role in the cognitive domain of tool-related actions, independently of the individuals’ handedness (Johnson-Frey et al., 2005; Króliczak et al., 2016).

Studies investigating brain activity in response to tool use performance in healthy individuals mainly included young participants. Gap of knowledge exists regarding the effect of age-related neural changes on tool use performance and how structural and functional brain alterations during the process of healthy aging affects the neural basis underlying the proper use of a tool. Elucidating age-related effects on these neural correlates would furthermore facilitate drawing parallels between brain activation patterns, underlying proper tool use in healthy individuals, and lesion analyses in patients, as brain damages associated with apraxic symptoms mostly occur in older people.

### Motor performance in aging

On a behavioral level, several aspects, linked to motor performance of an action, are affected by age-related changes. Worse spatial coordination of movements (Buckles, 1993; Contreras-Vidal et al., 1998), slower movement execution (Buckles, 1993; Rosjat et al., 2021), larger movement initiation phase (Frolov et al., 2020), higher co-activation of antagonistic muscles (Reuter et al., 2015) and increased variability (Diermayr et al., 2011) are, for example, observed in older compared to younger individuals. Moreover, a stronger dependence of accurate motor task performance on cognitive abilities is observed with increasing age (Maes et al., 2017) and the level of a task’s complexity plays an important role, particularly determining the amount of detectable age-related differences (Voelcker-Rehage, 2008). In addition, the asymmetry between dominant and non-dominant hand in motor behavior seems to decrease with age (Przybyla et al., 2011). Although several studies have focused on the identification of age-related effects on the behavioral level of human motor performance involved in a complex action, little is known about the underlying, age-related neural alterations (Seidler et al., 2010; Rosjat et al., 2021) and their impact on tool use performance.

### Structural and functional neural changes during healthy aging

On a neural level, with increasing age, structural and functional reorganization procedures take place (Bishop et al., 2010; Grady, 2012; Song et al., 2014; Fujita et al., 2023). Structural changes refer to the common finding of global gray matter volume reduction (Courchesne et al., 2000; Good et al., 2001; Smith et al., 2007) and alterations in white matter structures (Courchesne et al., 2000; Hedden and Gabrieli, 2004; Walhovd et al., 2011; Liu et al., 2017). Besides existing evidence for high interindividual variability in age-related volume changes (Fujita et al., 2023), the effects of healthy aging on structural brain characteristics are heterogeneous and non-uniform among different regions, leading to certain brain areas being more strongly affected by age-related, gray or white matter, structural changes than others (Raz et al., 1997; Salat et al., 2005; Raz and Rodrigue, 2006). Whereas high vulnerability of frontal and temporal regions to age-related effects is reported fairly consistent across multiple studies (Raz and Rodrigue, 2006; Fjell et al., 2014; Fujita et al., 2023), the effects of healthy aging on structural properties in other brain regions are more incongruent (Raz and Rodrigue, 2006; Walhovd et al., 2011). In addition, brain regions differ in their time point of experiencing accelerated volume loss (Fujita et al., 2023).

Referring to functional reorganization processes, studies focusing on the effects of aging on cognitive and motor task performance (Mattay et al., 2002; Ward and Frackowiak, 2003; Heuninckx et al., 2008) revealed less lateralization and higher degree of bilateral brain activation in older compared to younger individuals (Cabeza, 2002). According to Cabeza (2002) these age-related brain activation differences can be either linked to the individuals’ attempt to functionally compensate experienced neurocognitive decline, or to ongoing de-differentiation processes, indicating reduced task-specificity in brain activation patterns (Cabeza, 2002). Theoretically, this additional recruitment of bilateral, widespread brain regions is recapped by the HAROLD model, providing a framework for the observed phenomenon of hemispheric asymmetry reduction in older adults (Cabeza, 2002). Whereas a few neuroimaging studies, investigating brain mechanisms underlying motor performance, provide evidence for more widespread, bilateral and less lateralized brain activation in older compared to younger individuals (Mattay et al., 2002; Naccarato et al., 2006; Heuninckx et al., 2008), little is known whether these characteristics are also prominent during the performance of a complex, everyday action such as using a real tool.

Besides the impact of healthy aging on focal functional reorganization processes, healthy aging also affects large-scale brain networks and their dynamic interactions (Bergfield et al., 2010; Zhu et al., 2012; Song et al., 2014; Sala-Llonch et al., 2015). Regarding changes in brain networks’ connectivity during aging, decreased segregation of neural network, linked to reduced within-network and increased between-network functional connectivity is reported (Chan et al., 2014; Deery et al., 2023). Previous studies, investigating age-related connectivity differences, mainly addressed, within- and between functional connectivity of default mode network (DMN) (Song et al., 2014; Damoiseaux, 2017), a domain-general network, characterized by deactivation during task performance (Raichle, 2015; Sala-Llonch et al., 2015). Evidence indicates, that brain activation patterns in older individuals, are characterized by the reduced ability to flexibly suppress the default mode network’s activity during cognitive task performance (Spreng and Turner, 2019). On a behavioral level, the reduced ability to deactivate DMN during the performance of cognitively demanding tasks is associated with a stronger reliance on prior semantic knowledge (Spreng and Turner, 2019), which in turn plays a crucial role for the planning and preparatory phases of an action (Lindemann et al., 2006; Van Elk et al., 2008). Besides the DMN, further networks, including lateral frontoparietal regions (L-FPN), known as executive control network, are subjected to age-related changes (Li et al., 2015; Chand et al., 2017) and the dynamic relationship between DMN and L-FPN is associated with cognitive flexibility, an important executive function throughout the entire lifespan (Kupis et al., 2021). Past research, aiming at investigating the age-related effects on functional differences in DMN and L-FPN involvement, mainly addressed semantic abilities (Kupis et al., 2021; Martin et al., 2021). Gap of knowledge exists, whether the relationship between these networks is also valid for complex object manipulation tasks. Highly overlearned actions, like using a very familiar, common tool, involves the recruitment of semantic knowledge, such as the functional characteristic of the tool or the context in which the tool was used in the past (Silveri and Ciccarelli, 2009; Lesourd et al., 2016). Therefore, regions belonging to the DMN might take a larger role during planning, when reliance on prior semantic knowledge is stronger. Recalling semantic knowledge in these phases can include the context and the manner in which a presented object was used in the past, leading to action planning patterns, very similar to past experiences. These behavioral and neural characteristics of planning a complex object manipulation task, supposed to be strongly influenced by prior semantic knowledge, might further be related to minor possibility, implementing flexible adaptation during the actual task execution. Regions, such as the anterior cingulate cortex, the inferior parietal lobe or the dorsal prefrontal cortex, play a crucial role in providing flexible adjustments (Dosenbach et al., 2007), leading to the question how both networks (DMN and L-FPN) are involved in planning and executing a complex object manipulation task.

Based on this previous work, the aim of the current study is to investigate age-related changes in the neural tool use network, relevant for planning and executing real complex actions, as a fundamental component of everyday activities. Starting from findings of clear asymmetric brain activation during tool use in young adults (Johnson-Frey et al., 2005; Brandi et al., 2014), we planned to explore the theories of age-related bilateralization. Moreover, by investigating the default mode network (DMN), associated with recalling semantic knowledge (Spreng and Turner, 2019) during the planning phase, and its relationship with the executive control network (L-FPN), linked to flexible adaptation and cognitive control mechanisms (Cole et al., 2013) during movement execution, the current study aims to distinguish the role of these networks in different phases of tool use performance across age ranges. Gaining knowledge about these age-related changes and their further impact on neural networks, relevant for proper use of tools, provides an extended understanding of the neural basis underlying apraxia. Besides the effects of lesions, additional age-related neural changes could have significant impact on tool use performance and further represent relevant and confounding factors for apraxic symptom’s manifestation. We hypothesize that healthy aging affects the underlying neural basis of tool use performance and that with increasing age, a more widespread, bilateral recruitment of brain areas can be observed. Moreover, we speculate that both networks (default mode and executive control network) are differently involved in the two consecutives, planning and execution phases across age groups.

Following Brandi et al. (2014), a tool carousel within an fMRI experiment was applied. This setting enabled the acquisition of brain activation, underlying the performance of real tool use, in young and older adults.

## Materials and methods

Data of the younger cohort are part of the previous publication by Brandi et al. (2014). In the present study only usage of the left hand was analyzed. The same basic experimental set-up, procedure and methods of data analysis have been used in the current study. Both datasets were collected in close succession, by using the same scanning equipment and instrument. A short summary of the applied methods and approaches will be given here, however, for detailed information see the article published previously (Brandi et al., 2014). All changes, new and additional analysis, conducted in this study, can be found in the following sections.

### Participants

20 healthy older adults participated in the current study. Four individuals had to be excluded due to strong head movement (defined as head motions exceeding 3 mm in translation and 3° in rotation). The dataset of young adults, presented in this study, is overlapping with the data presented in the study published previously (Brandi et al., 2014). One participant belonging to the group of younger individuals was excluded randomly, in order to have a similar number of participants in both age groups. Thus, fMRI data from a balanced amount of 16 older (6 females) and 16 younger (5 females) individuals was included. Prior to the study, each participant provided an informed consent, which was approved by the local ethic committee. Participants in the older group had a mean age of 67.6 years (*SD* = 7.03, age range = 53–78 years). The mean age of the younger group was 25.4 years (*SD* = 1.88, age range = 21–28 years). All participants had normal or corrected-to-normal vision and showed no history of neurological or psychiatric disorders. The participants were all right-handed, measured with the Edinburgh Handedness Inventory (Oldfield, 1971).

### Experimental set-up and design

The so-called tool carousel (see Brandi et al., 2014) was used to present real, reachable, and usable stimuli to the participants. The study included three different experimental factors: the type of object the participants had to manipulate, the type of manipulation done with the object and the participant’s age.

The presented stimuli included ten different, familiar tools, used in everyday life and ten neutral objects. The set of familiar tools comprised a hammer, pencil, spoon, knife, lighter, bottle opener, key, screwdriver, tweezer, and scissor all made of MRI compatible material. In addition to the single tool, a tool-specific recipient was presented, which could be functionally manipulated with the tool (e.g., a rotatable screw for the screwdriver or a paper to write on with the pencil). Ten neutral objects were further chosen, with handles matching the actual tool in order to reduce visual and tactile differences. All neutral objects were bar-shaped and for simplification, we refer to this set of stimuli as “bars.” The bars were marked in blue on one end and could be placed in a blue marked opening on the workspace.

Two different types of manipulation had to be done with both sets of stimuli, indicated by a letter on top of the presented tool carousel compartment. Either an object had to be used (letter: B) or transported (letter: T). When tools were presented and had to be used, tools had to be grasped and handled according to daily life experiences (e.g., the key had to be turned in the keyhole or the hammer had to be moved up and down to hit the nail). When bars were presented and had to be used, according to the given instruction, the blue end of the bar had to be placed in the opening on the workspace. However, the left-or-right orientation of the blue end was altered across the experiment. Therefore, participants had to adjust their grip in order to place the bar into the opening comfortably (Rosenbaum and Jorgensen, 1992).

When tools or bars were presented and had to be transported, the objects had to be grasped and lifted before they were returned to the mounting. In total, this experimental set-up revealed four main conditions for both groups:

1. Tool use: a functional manipulation of a known object.
2. Tool transport: a non-functional manipulation of a known object.
3. Bar use: a functional manipulation of an unknown object.
4. Bar transport: a non-functional manipulation of an unknown object.

**FIGURE 1:**
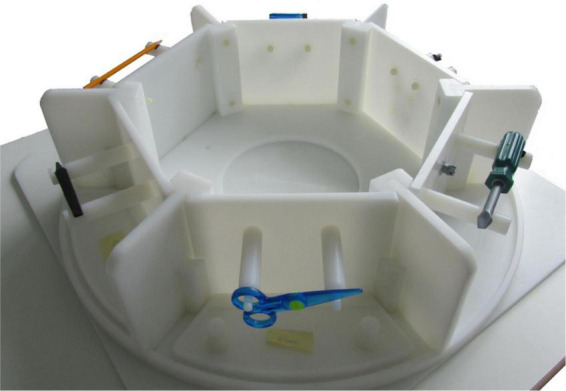
Representation of the tool carousel, including six compartments and mountings, whereas each compartment contains an object and its according recipient. The carousel was placed on a framework above the participants’ hip just outside the scanner core, while they themselves were lying in the scanner. Through a two-mirror system, participants had visual access to exactly one compartment. Tools could be conveniently manipulated with the outstretched arm. The examiner sat at the scanner bed, equipped the compartments with the upcoming tools and recipients and rotated the carousel upon an auditory command delivered via headphones from a control program.

Figure 1 shows the tool carousel equipped with some of the used tools and the corresponding recipients. However, for a detailed overview, see the article published previously (Brandi et al., 2014). The entire experiment consisted of 200 trials, divided into 160 condition trials and 40 control trials (no stimulus or task cue was presented). The experimental time-course was separated into two different phases: first, the planning phase, starting when the object and the cue for the task were visible to the participant; second, the execution phase, covering the actual movement of an action. A green light signaled the start of the 4 s execution phase, which began 2–6 s after the start of the planning phase. Participants were instructed to perform the execution task only when the green light switched on. This light switched on in 50% of all trials only. In the remaining 50% of trials no execution signal appeared and consequently subject had to pause. By implementing this Go-No-Go paradigm, analysis of pure planning phase without any movement related brain activation patterns was possible. The sequence of trials with green signal was random and the timing of the appearing signal varied between 2–6 s. Therefore, the experimental set-up did not allow to predict the onset of the green light and participants had to prepare and plan the following action always and whenever they saw the object. The execution phase was defined by the period in which the green light switched on and the actual movement occurred, captured by video recordings.

As testing two runs, according to the study published previously (Brandi et al., 2014), was too uncomfortable and exhausting for older participants, participants had to perform only one run, using the non-dominant, left hand. Choosing this hand allows to differentiate between brain activity involved in planning and executing object manipulation procedures, primarily expected in the ipsilateral, left hemisphere, and primary motor activity, expected in the contralateral, right hemisphere. Moreover, as most patients with apraxic symptoms, show left hemispheric lesions, further linked to right-sided paralysis, results of the current study can be incorporated better into apraxia research.

### Procedure

Before starting the experiment, all participants had to answer a questionnaire, referring to their knowledge and familiarity with the usage of presented objects. The maximum score testing tool knowledge was 70 points (max. 7 points for each object), indicating that all tools were used daily during the participant’s job and routines. On the contrary, the minimum score of 0 would mean, that the object has never been used before and participants were not familiar with its usage. Possible differences of obtained scores between both groups were calculated by applying Mann-Whitney-U-Test. Video instructions were used for the explanation of the experimental tasks and cues. Moreover, all conditions and tasks were trained within the scanner, until the participants were familiar with the tasks and capable to perform them. The Presentation Software (Neurobehavioral Systems, Inc., Berkeley, CA^1^) was used to give acoustic instructions via headphones to the operator in the scanning room. For each trial, the task cues and stimuli were exchanged by the operator and the tool carousel was turned on the acoustic cues. A camera recorded the workspace and hands of the participants during the entire experiment for evaluation of behavioral outcomes.

### Video analysis

In order to capture and evaluate the individuals’ performances, errors were counted. Three error types were differentiated:

1. General task error: The task was not performed according to the cue.
2. Grip error: The objects were not grasped according to the instructions or daily routines.
3. Movement error: The movement of the action was not performed completely or according to the instructions.

Mann–Whitney-U-Test was calculated in order to measure inter-group differences of the errors made. Additionally, the acquired video data was analyzed with a motion detector (MATLAB). This analysis allowed to evaluate the participant’s duration of each movement, thus the reaction times between the onset of a green light and the start of a movement. Two independent *t*-tests were calculated in order to measure inter-group differences of reaction time and movement duration.

### MRI measurement

The MRI measurements were performed on a Siemens (Erlangen, Germany) 3 Tesla Verio MRI scanner. T1- weighted anatomical images were acquired with the MPRAGE (magnetization-prepared rapid acquisition gradient echo) sequence. The BOLD (blood oxygenation-level-dependent) echo-planar images were measured using a T2*- weighted gradient echo sequence with the repetition time TR = 2,000 ms, echo time TE = 30 ms, FoV (field of view) = 192 mm, flip angle α = 90°, matrix = 64 × 64, slices = 35, slice thickness = 3 mm and voxel size = 3 mm × 3 mm × 3 mm.

### fMRI data analysis

The entire data analysis was performed with SPM12 (Ashburner et al., 2014). Further, the CONN Toolbox (Whitfield-Gabrieli and Nieto-Castanon, 2012) was used in order to preprocess the fMRI data. The default preprocessing pipeline for volume-based analysis (direct normalization to MNI space) was applied, including realignment, slice-time correction, normalization to MNI space and smoothing procedures. An 8 mm Gaussian Kernel (FWHM) was applied in order to smooth the functional data. The data analysis was first done on a single subject, first level, and calculated contrasts were then entered into a group, second level factorial analysis. On the first level, a general linear model (GLM) was specified for each subject, including a total amount of 15 regressors. A separate regressor for the planning phases [all No-Go conditions (not followed by a green action signal appearance), and the planning phase of all Go conditions (followed by a green action signal appearance)] and the execution phase, for each of the four conditions (tool use, tool transport, bar use, bar transport), was set up, yielding a total amount of 12 regressors. As a separate regressor for the movement duration entered the GLM, all experimental events were modeled, purely including their onset times. Two regressors for the control condition were specified, one covering the duration of all movements and the other one modeling the errors made by participants individually. In order to ensure that differences between the conditions were not caused and confounded by differences in the duration of movement execution, a regressor was further entered, accounted for the duration of all movements.

Additionally, the realignment movement parameters were entered into the model as regressors of no interest in order to control for any motion related artifact. Based on the output of individual realignment procedure, scan-to-scan movement was calculated. Trials in which scan-to-scan movement exceeded a threshold of 3 mm in any direction were identified and entered the individual GLM as errors. Thus, in the group of older participants a total amount of 25 trials, in the group of younger participants only one trial, had to be excluded.

On a first level, all conditions were contrasted against the intrinsic baseline. The resulting contrast images further entered the group-based, second level analysis. The model on the second level was analyzed separately for the planning and execution phase in a 2 × 2 × 2 factorial design including the factors object (tool and bar), task (use and transport) and group (older and young adults). The main effects for the factors object and task for each group individually, and the main effect for the factor group were of most interest. The Automated Anatomical Labeling (AAL) atlas (Tzourio-Mazoyer et al., 2002) was used in order to label the activated brain areas. The graphical displays were created with BrainNet viewer (Xia et al., 2013). Throughout the entire SPM analysis, a statistical threshold of *p*_*cl*_ < 0.05 with underlying voxel-based threshold of *p_*unc*_* < 0.001 was applied.

### Laterality index

Besides the conduction of conventional SPM analysis, the purpose of the current study was the investigation of differences in the lateralization of brain activation patterns in older compared to younger individuals. Lateralization of the involved brain regions was tested in order to investigate, whether the current dataset supports the HAROLD model (Cabeza, 2002). By applying LI toolbox (Wilke and Lidzba, 2007) (settings: bootstrapping method, inclusive mask for occipital, temporal, parietal, frontal lobe; exclusive mask midline ± 5 mm), laterality index (LI) was calculated for each subject and each condition, separately. A *t*-test with Bonferroni correction was further performed for each group in order to evaluate whether the LIs differ significantly from 0 (LI of 0 indicates symmetrical activation pattern, LI > 0 indicates left-sided lateralization, LI < 0 indicates right-sided lateralization). The LI analysis was performed for frontal, parietal, temporal and occipital lobes, respectively. An ANOVA was further conducted, including groups, tasks, lobes as main and groups*tasks and groups*lobes as interaction effects, in order to test for any influencing factors on the amount and direction of lateralization.

### Involvement of default mode and executive control network

MarsBaR toolbox was used in order to build a mask for each network, assuming to be subjected to age-related changes. Peak centroid MNI coordinates were extracted for the lateral frontoparietal network (L-FPN) (Dajani et al., 2020) and the default mode network (DMN) (Schaefer et al., 2018), adding around a sphere of 6 mm, respectively. As we were particularly interested in left-hemispheric activation, the L-FPN network included the dorsal anterior cingulate cortex, the left dorsolateral prefrontal cortex, anterior insula and inferior parietal lobe. Those regions are reported fairly consistent with regards to their role in cognitive flexibility tasks (Leber et al., 2008; Dajani and Uddin, 2015; Dajani et al., 2020). In order to investigate the involvement of DMN and L-FPN in complex object manipulation task, beta-weights from the previous GLM analysis of DMN regions during planning, and beta-weights of L-FPN regions during execution were extracted. Those extracted beta-weights then entered a Pearson correlation analysis. As we were particularly interested in the relationship of these networks, being involved in tool use performance, beta-weights were purely extracted for the tool use and the bar use, control condition. The masks and according MNI centroid coordinates, being applied to extract the according beta-weights for DMN and L-FPN involvement, are presented in the Supplementary material.

## Results

### Behavioral data: questionnaires

The scores of the behavioral data and according *p*-values of the conducted group comparisons are listed in Table 1. Data indicate, that both age groups knew all tools and were similarly familiar with their usage, while both groups did not know, nor were familiar with the presented bars, used as control condition within the experiment.

**TABLE 1:** Demographic information and results of behavioral data.

	Older participants	Younger participants
**Demographic information and behavioral scores**		
*N*	16	16
Gender (female,%)	40	33.3
Age (y), *M (SD)*	67.6 (7.03)	25.4 (1.88)
Handedness	100 (70–100), *p* = 0.5^1^	95 (70–100)
Movement duration (s), *M (SD)*	4.14 (0.45), *p* = 0.65^1^	4.11 (0.49)
Reaction time (s), *M (SD)*	0.56 (0.19), *p* = 0.16^1^	0.52 (0.10)
**Tool related knowledge**		
Tool knowledge	70 (66–70), *p* = 0.43^2^	70 (66–70)
Tool familiarity	98 (68–125), *p* = 0.11^2^	94 (73–111)
Score neutral object	0 (0–2), *p* = 0.8^2^	0 (0–2)
**Errors**		
Grip errors	1.5 (0–3), *p* = 0.01*^2^	0 (0–3)
Movement errors	1 (0–3), *p* = 0.12^2^	0 (0–3)
Task errors	1 (0–6), *p* = 0.02*^2^	0 (0–2)
Total errors	4 (0–7), *p* = < 0.001*^2^	1 (0–5)

Familiarity and knowledge about the tools: the range of all scores is given in parentheses behind the median. A star (*) indicate significant *p*-values at α < 0.05.

^1^represent *p*-values of independent *t*-test.

^2^represent *p*-values after group-wise comparison of median values between both age groups.

### Behavioral data: video analysis

The participants’ performance, the movement duration and the reaction times were captured by evaluating the acquired video sequences. Inter-group comparisons were further calculated, including these behavioral outcomes. The median error scores of the grip, movement and task errors and the total amount of errors along with the according *p*-values of inter-group comparisons are listed in Table 1. The older individuals made significantly more grip and task errors compared to younger adults, further resulting in a significantly higher total error score. The results of the motion detection analysis, including movement duration and reaction times, are also depicted in Table 1. Table 1 shows that movement duration as well as reaction times did not differ significantly between both age groups. Based on trial exclusion after scan-to-scan movement calculation, more trials had to be excluded in older (25 trials in total, <1% of all trials executed by the group of older participants) compared to younger individuals (1 trial in total).

### fMRI data: within-group analysis

**FIGURE 2:**
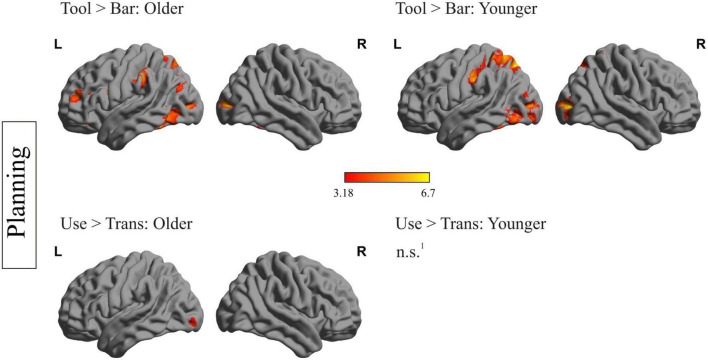
Within-group differences during the planning phase: depiction of within-group differences during the planning phase for the main factor object **(upper row)** and the main factor task **(lower row)**, for the older **(left side)** and the younger participants **(right side)**. ^1^No region survived the applied threshold; *p_*cl*_* < 0.05, with underlying voxel-threshold of *p*_*unc*_ < 0.001.

**FIGURE 3:**
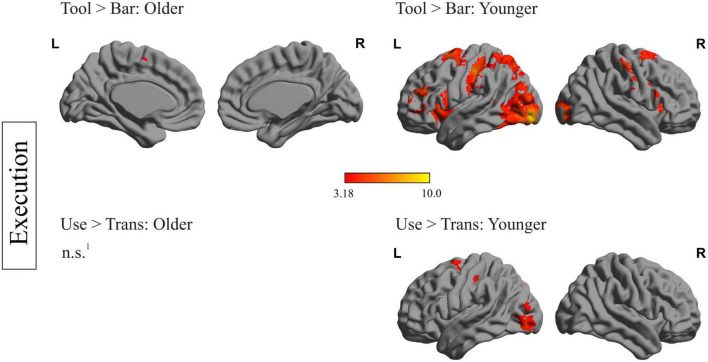
Within-group differences during the execution phase: depiction of within-group differences during the execution phase for the main factor object **(upper row)** and the main factor task **(lower row)**, for the older **(left side)** and the younger participants **(right side)**. ^1^No region survived the applied threshold; *p*_*cl*_ < 0.05, with underlying voxel-threshold of *p*_*unc*_ < 0.001.

Within-group analyses for older and younger participants during planning and execution was conducted. In order to identify brain regions relevant for planning and executing a complex action, the main effects for the factors object and task were calculated by comparing the conditions tool > bar and use > transport for the planning and execution phase and for both age groups, separately. The resulting heat maps are displayed in Figures 2, 3, presenting the brain maps of the older individuals on the left-hand and those of the younger individuals on the right-hand side for the two mentioned contrasts.

### fMRI data: within-group analysis during planning phase

Figure 2 represents the within-group differences during the planning phase. Regarding the main effect of the factor object (tool > bar) in older individuals, a mainly left-lateralized wide network, including frontal, temporal, parietal and occipital regions was identified. Younger participants showed significantly higher activation in left-sided temporal, parietal and occipital regions during the planning phase of tool compared to bar. Importantly, younger individuals did not show stronger activation of frontal areas when a tool- compared to a bar- related action was planned. Regions, that were more strongly activated during the planning phase of a tool- compared to a bar-related action, in both age groups, are the left inferior temporal gyrus, the left middle occipital gyrus, the left inferior occipital gyrus, the left parietal superior gyrus and the left precuneus. The left supramarginal gyrus, the left inferior and middle frontal gyrus were solely activated in older participants when they were faced with a tool compared to a bar.

Regarding the main effect of the factor task (use > trans) during the planning phase, no brain region in younger individuals survived the applied threshold. In older individuals, the left precentral gyrus, the left inferior and middle occipital gyrus as well as right hemispheric cerebellar regions were more activated when an object had to be used compared to transported.

A complete list containing the statistics of activated brain regions, the according peak MNI- coordinates and *p*-values, is presented in the Supplementary material.

### fMRI data: within-group analysis during execution phase

Figure 3 represents the within-group differences during execution. Regarding the main effect of the factor object (tool > bar), only the left and right supplementary motor area as well as the left middle cingulate gyrus survived the applied threshold in older participants. Younger participants, however, showed significant stronger activation in frontal (left middle and inferior frontal gyrus, right postcentral gyrus), temporal (right superior temporal gyrus), as well as occipital (left occipital middle gyrus, right occipital inferior gyrus) and subcortical areas (right putamen and insula) when a tool- compared to a bar-related action was executed. Regarding the main effect of the factor task (use > trans) no region survived the applied threshold in older participants. In younger participants this comparison revealed stronger activation in left-hemispheric superior frontal gyrus, middle occipital gyrus and postcentral gyrus.

A complete list containing the statistics of activated brain regions, the according peak MNI- coordinates and *p*-values, is presented in the Supplementary material.

### fMRI data: between-group analysis

**FIGURE 4:**
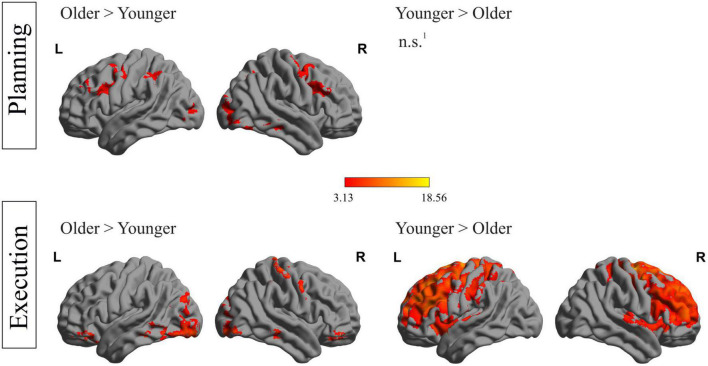
Between-group differences during both phases: depiction of between-group differences for the planning phase **(upper row)** and the execution phase **(lower row)**. ^1^No region survived the applied threshold; *p*_*cl*_ < 0.05, with underlying voxel-threshold of *p*_*unc*_ < 0.001.

Besides within-group analyses including the main factors objects (tool > bar) and tasks (use > trans), additional between-group analyses were conducted, comparing whole-brain activation during all planning and execution phases between older and younger individuals, respectively. Results are presented in Figure 4. During the planning phases, several frontal (left precentral and right middle frontal gyrus), temporal (right middle and inferior temporal gyrus), parietal (left and right inferior parietal and angular gyrus) and occipital (left middle occipital gyrus) regions were significantly stronger activated in older compared to younger individuals. No region could be identified, which was activated stronger in younger than older individuals.

Figure 4 (lower row) shows differences in activation strength between both groups in the execution phase. Older compared to younger individuals showed stronger activation during execution in various areas, including frontal (right frontal superior gyrus, left posterior orbital gyrus, right anterior orbital gyrus), temporal (left inferior temporal gyrus), occipital (right lingual gyrus) and cerebellar regions. A stronger activation in younger compared to older participants during the execution phase was observed in a huge cluster, containing 68,838 voxels, with peak-coordinates in subcortical, caudate structures and extending to frontal and parietal regions. A complete list containing the statistics of activated brain regions, the according peak MNI- coordinates and *p*-values, is presented in the Supplementary material.

### Laterality index

**FIGURE 5:**
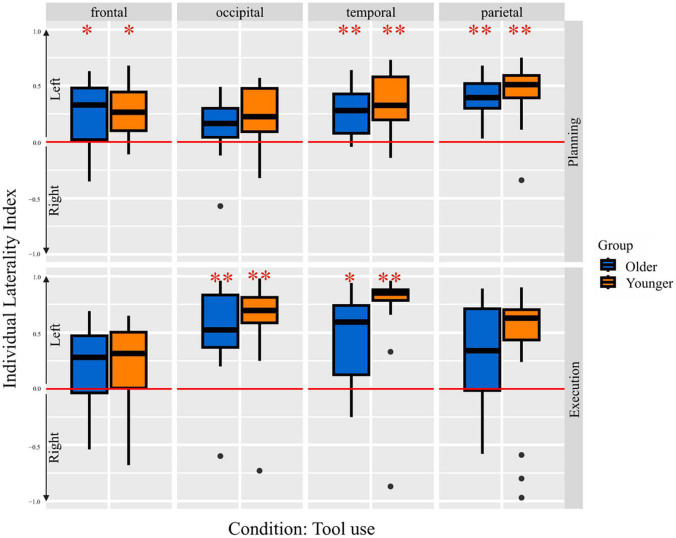
Laterality indices per group for both the planning and the execution phase for pure tool use condition. One-sample *t*-test results are indicated, *p < 0.05 and **p < 0.001, respectively.

In Figure 5 the mean LIs of both groups, for each brain region during the planning and execution phase of the tool use condition are depicted. One sample *t*-tests with Bonferroni correction revealed several lobes differing significantly from 0, thus indicating a significant lateralization effect. A positive LI value indicates left-, a negative LI value indicates right-lateralization. Older as well as younger adults showed left-lateralization in frontal, temporal and parietal lobe. Referring to tool use trials in the execution phase, older and younger adults showed left-lateralized activation in occipital and temporal lobes. A further conduction of 2 × 8 × 4 ANOVA (Groups, Conditions, Lobes) revealed a main effect for lobes [*F*(3,989) = 8.22, *p* = < 0.001] and tasks [*F*(7,989) = 4.61, *p* < 0.001]. Tukey HSD *post-hoc* test revealed higher mean LI values for temporal (*p*_*adj*_ < 0.001, 95% CI = [0.07;0.25]) and for occipital lobes (*p*_*adj*_ = 0.001, 95% CI = [0.04;0.21]) in contrast to the frontal lobe. The main effect of the task revealed a higher left-lateralization level for tool use execution condition compared to bar use (*p*_*adj*_ < 0.001, 95% CI = [0.07;0.37]) and bar transport planning condition (*p*_*adj*_ = 0.002, 95% CI = [0.04;0.34]), as well as higher left-lateralization level for the bar use execution condition compared to the bar use planning condition (*p*_*adj*_ = 0.010, 95% CI = [0.02;0.32]). There was no group effect observable, indicating equal amount of hemispheric asymmetry and left-lateralized activation across both groups.

### Default mode and executive control network

**FIGURE 6:**
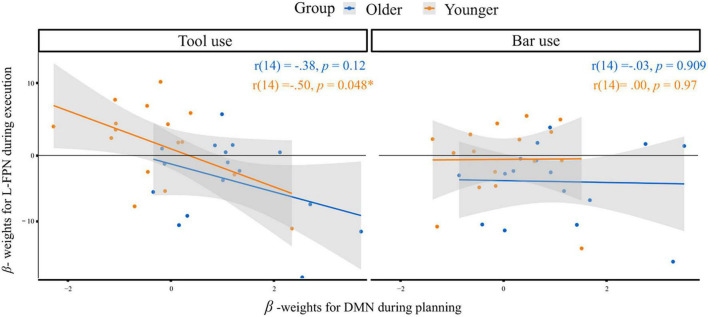
Correlation of extracted beta-weights for DMN regions during planning and L-FPN regions during execution for both groups in response to the tool use **(left)** and bar use **(right)** condition. Significant correlation at α < 0.05 is indicated*. *r* = Pearson-product-moment-correlation-coefficient.

As described in detail in the method section, further analyses aimed at investigating differences in the default mode network (DMN) involvement during planning and the executive control network (L-FPN) involvement during execution. In Figure 6 the correlations between the extracted beta- weights for the according regions and phases are depicted, for tool use and bar use condition. The bar use condition revealed nearly no correlation between DMN involvement during planning and L-FPN involvement during execution in older [*r*(14) = −0.03, *p* = 0.909] as well as younger [*r*(14) = 0.00, *p* = 0.97] individuals. The tool use condition revealed a significant negative correlation in younger subjects with lower beta-weights in DMN activation during planning being associated with higher beta-weights in L-FPN activation during execution and vice versa [*r*(14) = −0.50, *p* = 0.048]. The same correlation in older individuals did not reach the threshold of statistical significance [*r*(14) = −0.38, *p* = 0.12]. Nevertheless, the relationship between the networks being activated during planning and execution in older individuals for tool use revealed a regression slope similar to the one of young individuals and a medium effect size (Cohen, 1992).

## Discussion

In the current study we investigated functional changes during healthy aging and their impact on the neural basis, underlying planning and execution of complex object manipulations. Overall, our results indicate that the main activation pattern for the manipulation of tools compared to neutral objects (bars) was stable across age ranges and similar frontal, temporal, parietal and occipital regions were activated during the entire task. However, for the consecutive phases (planning and execution), our results showed stronger frontal, temporal, occipital and parietal engagement during the planning phases in older compared to younger adults. Moreover, our data revealed, that the neural correlates, supporting complex object manipulations, are mainly left-lateralized across both groups, concluding no age-related asymmetry reduction effect. On a behavioral level, neither reaction time nor movement duration differed significantly between groups. However, both groups differed in the amount of errors made and significantly more grip and task errors were made by older individuals.

To our knowledge, this is the first study comparing brain activity in response to real reachable objects between older and younger participants within an fMRI task-related experiment. The activated brain regions during the planning phase in older individuals are consistent with previous literature, indicating the praxis representation network, being already and mostly involved in planning a tool-related action (Frey, 2008; Gallivan et al., 2013; Michalowski et al., 2022). Importantly, frontal regions, essentially the left middle and inferior frontal gyrus, were strongly activated in planning tool-related compared to bar-related actions in older but not younger individuals. This age-related increase of frontal activity during the planning phase in older compared to younger adults, leads us to assume that executive functions are strongly involved in the action preparation phases, similar to the obtained results by Berchicci et al. (2012). As we had only one performance measure (errors made) at the end of the entire task, our experimental set-up does not allow to clarify if errors were already made during the planning phase of the task. Therefore, the findings of our study, (stronger activation during planning but not necessarily during execution in older individuals), cannot be related to performance measures, purely related to these specific phases. Thus, conclusions about any compensatory mechanism are challenging.

### Shift from execution to planning in older individuals

A central finding of our study revealed a stronger investment into the planning phase of a complex object manipulation task in older compared to younger individuals. We interpret this finding as an age-related difference in the strategy used. Whereas older individuals tend to behave more proactively, younger individuals apply more online-control strategies, implemented in reactive behavior. This finding is partly inconsistent with previous neuroimaging studies, indicating an opposite switch from proactive to reactive control with increasing age in task-switching environments (Jimura and Braver, 2009; Kopp et al., 2014). Also Wunsch et al. (2017) reported a decline of anticipatory motor planning skills in older people. However, consistent with our results, Frolov et al. (2020) reported prolonged movement execution phase in older individuals, further related to increased utilization of preparatory processes, preceding the actual movement execution phase. Based on our understanding, the planning and execution phases differ in the cognitive as well as perceptive processes being involved. During the planning phase, the object must be perceived sensory, semantic knowledge about function might be retrieved, the goal of the consecutive action must be set up und finally the motor plan must be prepared. During the execution phase, however, the motor plan prepared previously, needs to be implemented and carried out, further monitored, and controlled in order to achieve the goal-directed movement. Our results indicate that the planning phase of complex actions was highly important for older individuals, consistent with the observation that age-related effects can be already seen within the movement preparation and planning phase and are not purely restricted to the time-frame in which an actual movement is carried out (Reuter et al., 2015).

Moreover, our results point toward the importance of analyzing and characterizing different temporal, consecutive phases. To our knowledge there is only one previous event-related fMRI study, distinguishing planning and execution phase in a tool-oriented pantomime task (Michalowski et al., 2022). Similar to our results, the authors concluded the mainly left-lateralized praxis representation network being already involved in the planning phase of an action, and the more demanding the task, the more right-hemispheric regions were additionally activated. However, as young healthy individuals were included (Michalowski et al., 2022), the classification of our results with regard to the age differences reported in previous literature still remains challenging. Berchicci et al. (2012) conducted an EEG study, investigating the age-related effects on cortical activity in response to reaction time tasks. Similar to our results, the authors detected a shift from the stimulus processing to the motor planning stage in older adults, interpreted as an applied strategy in order to achieve the same accuracy rate as younger adults (Berchicci et al., 2012).

The shift from pursuing a flexible online-control strategy during the action execution, to a stronger focus on the preparatory, planning phase, which was only observable in the older aging group, leads us to assume that due to limited cognitive and neural resources, the applied effort in order to plan the consecutive task, could not be maintained across the entire experimental task. Thus, older participants might experience a trade-off, leading to devote their limited resources rather for the planning than the execution part of such a complex action. The intense investment into the planning phase might be associated with the stronger reliance on prior semantic knowledge and its neurobiological basis of DMN involvement (Spreng and Turner, 2019). An additional analysis, correlating DMN activity during planning and L-FPN activity during execution revealed that coupling mechanisms between both networks were more prominent in tool use compared to bar use conditions in both groups. Although, the negative correlation between the networks revealed significance only in younger adults, older adults showed similar relationships of involved networks with medium effect size (Cohen, 1992). Furthermore, on a descriptive level, a shift of DMN involvement in older individuals toward positive values of extracted beta-weights could be observed, indicating higher activation (reduced deactivation) during the planning phase in older individuals. On a behavioral level, this reduced deactivation of DMN might be linked to recall and focus on semantic knowledge (Spreng and Turner, 2019) as well as past tool-related experiences during planning, combined with lower possibility to behave cognitively flexible during the actual movement execution. This assumption can be linked to an fMRI study published previously, focusing on the examination of DMN involvement during a cognitive flexibility task (Vatansever et al., 2017). The authors concluded the DMN being involved in making predictions, based on gained knowledge during past experiences, whereas control networks come into play, when discrepancies exist between predictions made previously and perception of the current situation. We speculate that the shift toward the planning phase observed in older individuals is somehow related to the stronger involvement of semantic knowledge, an ability weakly influenced by age-related changes (Spreng and Turner, 2019). Additionally, the influence of predictions, made in the movement preparation phase and again linked to memory recall (Vatansever et al., 2017), could further contribute explaining the observed shift in older individuals. Future studies should elaborate on these age-related differences in large scale network recruitment.

### No effect of aging on lateralization

Our data does not support the hypothesis of age-related hemispheric asymmetry reduction during complex object manipulation tasks. Referring to this aspect, we see parallels between our results and those investigating age-related effects on lateralization during tasks testing semantic and language abilities. These studies reported no age-related effect on the neural correlates involved in semantic ability tasks, resulting in similar lateralization patterns and core brain regions recruited by older as well as younger individuals (Kennedy et al., 2015; Hoffman and Morcom, 2018). Given the fact that semantic and language abilities are related to tool use performance (Higuchi et al., 2009; Silveri and Ciccarelli, 2009; Thibault et al., 2021), our results can be linked to these studies of cognition semantics.

The conducted ANOVA, including individual laterality indices, revealed a main effect for occipital and temporal lobes, showing significantly stronger left-lateralization compared to the frontal lobe. This finding is probably due to the use of the left-hand, associated with right-hemispheric motor area involvement and thus, revealing additional right-hemispheric contribution of frontal areas, besides the left-lateralized frontal regions, as part of the praxis representation network (Gallivan et al., 2013; Buxbaum et al., 2014; Michalowski et al., 2022). Additionally, the conducted ANOVA revealed a main effect for the factor task. Thus, execution of the tool use task revealed significantly stronger left-lateralization compared to the planning phase of the bar use and bar transport condition, emphasizing the significance of the left-lateralized, tool specific, praxis representation network for proper tool-related actions performance. Moreover, the stronger left-lateralization during the tool use execution condition with the non-dominant left hand confirms the importance of left-hemispheric contribution and functioning for proper tool use performance, besides the right-hemispheric activation, enabling left-hand usage. Another important finding of ANOVA calculation consists in the absence of any age-related lateralization effect, leading to the conclusion that left-hemispheric neural activity is equally relevant for older as well as younger individuals. Thus, our data, regarding brain activation in response to tool use performance, does not support the HAROLD model (Cabeza, 2002). Considering the shared mechanisms between tool use and language performance (Higuchi et al., 2009; Thibault et al., 2021), our result is reminiscent of brain activation in response to a semantic ability task, remaining also left-lateralized across age groups (Nenert et al., 2017).

However, between-group comparisons, as depicted in Figure 4, allowed for a deeper look at lateralization patterns during all planning phases, no matter which object had to be manipulated in which form. This group-wise comparison revealed additional right hemispheric activation of frontal, occipital, temporal and parietal areas in older people during the planning phases of a complex object manipulation task. The bilateral brain activation in older people could be related to compensation of tool use deficits, which could further manifest in correlation differences between the activation strength and the performance measure across both age groups. However, our error analysis was influenced by the experimental set-up, resulting in one overall performance measure, comprising the entire trial. Thus, our experimental procedure did not enable us to correlate activation strength within the phases to the errors, made explicitly and purely during planning and execution. For clarification, a further adjustment of the experimental set-up would be needed.

### Behavioral differences

Referring to the behavioral outcome differences between younger and older participants, movement duration was similar across groups, however, accuracy of task performance was worse in older individuals. The speed-accuracy trade-off represents a relatively robust finding in aging literature, however, most older people tend to work slower, while maintaining the same accuracy rate (Salthouse, 1979; Forstmann et al., 2011). In contrast, our data suggest that older individuals do not sacrifice speed but are significantly more inaccurate in task processing. Although previous research already revealed that motor speed differences between age-groups do not mediate the observed deterioration of tool use skills (Lesourd et al., 2016), our finding, suggesting a favor to the task speed and at the expense of the task accuracy, might be due to the special conditions and settings of the task. While older people were similarly familiar and knew the presented tools quite well, they might have assumed, the tool could have been used during the scan according to their past daily life experiences, leading to similar reaction times within the first phase of the task. However, while lying and having lower possibility of moving arm and hand than in real life, grasping the tool within this fMRI setting might have needed an adjusted technique. More difficult adjustment in the older participants may have resulted in higher error rates at the final stage of the tasks‘ fulfillment. Moreover, behavioral findings of age-related differences might additionally be confounded by the testing situation itself, performance expectancies, difficulties in implementing the given instructions (Desrichard and Köpetz, 2005) or impairments in sensory perception (Füllgrabe, 2020).

## Conclusion

In summary, our data showed, that brain regions involved in complex object manipulation remain stable across age-ranges. A wide left-lateralized network including similar frontal, parietal, temporal and occipital regions represents the neural basis, involved in tool use performance in both study groups. However, an important finding of our data points to the differences in the neural investment on both, the planning and execution phases, between groups. Whereas older people put more effort into the planning and preparation part, younger people seem to apply more online-control strategies during the actual movement execution phase. Moreover, our data does not support the hemispheric asymmetry reduction phenomenon, often reported in older individuals, indicating no age-related bilateralization of the neural basis underlying tool use performance. In order to gain a more holistic understanding of the contributing regions, widely distributed across the entire brain, further studies, addressing functional and effective connectivity differences across age groups, could provide important insights.

## Limitations

Several limitations must be considered when interpreting the results. Due to our small sample, there may be statistical power issues and problems of reproducing results obtained consistently (Grady et al., 2021). Additionally, differences in significant brain activation of functional data between young and older individuals can be confounded by multiple factors. First, differences in anatomical brain composition, characterized by local atrophy in older people, can influence functional data (Kalpouzos et al., 2012). Moreover, changes in vascular structure, neurovascular coupling and cerebral blood flow might represent confounding factors when interpreting the measured BOLD signal (D’esposito et al., 2003; Samanez-Larkin and D’esposito, 2008). Also an often observed lower signal to noise ratio (SNR) (Huettel et al., 2001) and higher motion-related artifacts in older compared to younger people could have influenced the results (Kannurpatti et al., 2010). Although the number of excluded trials, based on the applied motion criteria, was relatively low in both groups, older participants were observed to move more than younger in the remaining trials. Thus, a moderate bias due to motion-related artifacts in older individuals cannot be excluded. To conclude, significant differences in BOLD signal between younger and older individuals, can eventually not be exclusively interpreted as differences in neural activity (West et al., 2019). Since important effects are comparable in both age groups and some results manifest as a switch in activation (between planning and execution) and not in a general attenuation or increment of activity we believe that most of our results are largely robust against the above-mentioned sources of bias.

## Data availability statement

The raw data supporting the conclusions of this article will be made available by the authors, without undue reservation.

## Ethics statement

The studies involving humans were approved by the Ethics Committee of the Technical University of Munich. The studies were conducted in accordance with the local legislation and institutional requirements. The participants provided their written informed consent to participate in this study. Written informed consent was obtained from the individual(s) for the publication of any potentially identifiable images or data included in this article.

## Author contributions

CS: data analysis, literature review, and drafting manuscript. JZ: data analysis. M-LB: data collection and analysis and study-design. TK: literature review. AW and JH: conceptualization, study-design, and manuscript revision. All authors contributed to the article and approved the submitted version.

## Footnotes

1
www.neurobs.com
