# Supplementary material for: Investigating the effects of the aging brain on real tool use performance—an fMRI study
Source: Front Aging Neurosci. 2023 Aug 22;15:1238731. doi: 10.3389/fnagi.2023.1238731 (PMC10477673; doi:10.3389/fnagi.2023.1238731)
Supplement: Supplementary file 1 [file Data_Sheet_1.docx]

# Supplementary material

## Appendix A: Detailed description of activation statistics: cluster- and voxel level

| Contrast | Peak voxel coordinates in MNI space | | | *p_FWE corr_* | Cluster size | T value | *p_unc_* | AAL Labeling | Percentage of overlapping |
| --- | --- | --- | --- | --- | --- | --- | --- | --- | --- |
|  | X | Y | Z |  |  |  |  |  |  |
| Tool > Bar (Planning): Elderly | -48 | -56 | -14 | <.001 | 1510 | 6.21 | <.001 | L Inferior Temporal Gyrus | 67.18% |
|  |  |  |  |  |  |  |  | L Fusiform Gyrus | 21.55% |
|  | -34 | -84 | 0 |  |  | 5.63 | <.001 | L Middle Occipital Gyrus | 72.43% |
|  |  |  |  |  |  |  |  | L Inferior Occipital Gyrus | 25.05% |
|  | -44 | -60 | -6 |  |  | 4.98 | <.001 | L Inferior Temporal Gyrus | 29.32% |
|  |  |  |  |  |  |  |  | L Middle Temporal Gyrus | 25.83% |
|  |  |  |  |  |  |  |  | L Inferior Occipital Gyrus | 15.34% |
|  | -44 | 46 | 16 | <.001 | 283 | 5.75 | <.001 | L Middle Frontal Gyrus | 56.31% |
|  |  |  |  |  |  |  |  | L Inferior Frontal Gyrus, triangular part | 23.11% |
|  | -40 | 44 | 26 |  |  | 3.89 | <.001 | L Middle Frontal Gyrus | 81.75% |
|  | -34 | 44 | 36 |  |  | 3.87 | <.001 | L Middle Frontal Gyrus | 50.87% |
|  |  |  |  |  |  |  |  | L Frontal Superior Gyrus | 16.31% |
|  | 28 | -92 | 6 | <.001 | 459 | 5.75 | <.001 | R Middle Occipital Gyrus | 60.58% |
|  |  |  |  |  |  |  |  | R Superior Occipital Gyrus | 14.56% |
|  | 22 | -88 | -20 |  |  | 4.59 | <.001 | R Cerebellum Crus 1 | 38.45% |
|  |  |  |  |  |  |  |  | R Lingual Gyrus | 22.72% |
|  | 24 | -88 | -8 |  |  | 3.37 | .001 | R Lingual Gyrus | 48.54% |
|  |  |  |  |  |  |  |  | R Inferior Occipital Gyrus | 27.18% |
|  |  |  |  |  |  |  |  | R Fusiform Gyrus | 12.82% |
|  | -60 | -32 | 40 | <.001 | 512 | 5.19 | <.001 | L Supramarginal Gyrus | 36.89% |
|  |  |  |  |  |  |  |  | L Inferior Parietal Gyrus | 32.82% |
|  | -58 | -34 | 50 |  |  | 3.85 | <.001 | L Inferior Parietal Gyrus | 40.19% |
|  | -52 | -38 | 56 |  |  | 3.79 |  | L Inferior Parietal Gyrus | 33.79% |
|  |  |  |  |  |  |  |  | L Postcentral Gyrus | 25.24% |
|  | -14 | -74 | 60 | <.001 | 1160 | 5.12 | <.001 | L Precuneus | 30.87% |
|  |  |  |  |  |  |  |  | L Superior Parietal Gyrus | 22.14% |
|  | -8 | -68 | 62 |  |  | 5.05 | <.001 | L Precuneus | 65.83% |
|  | -18 | -64 | 54 |  |  | 4.52 | <.001 | L Parietal Gyrus | 75.15% |
|  |  |  |  |  |  |  |  | L Superior Precuneus | 24.66% |
|  | -46 | 8 | 18 | <.001 | 181 | 4.75 | <.001 | L Inferior Frontal Gyrus, opercular part | 64.47% |
|  |  |  |  |  |  |  |  | L Precentral Gyrus | 17.28% |
|  |  |  |  |  |  |  |  | L Rolandic Oper | 13.2% |
|  | -46 | 6 | 32 |  |  | 4.03 | <.001 | L Precentral Gyrus | 70.29% |
|  |  |  |  |  |  |  |  | L Inferior Frontal Gyrus, opercular part | 19.22% |
|  | -24 | 30 | -18 | .017 | 82 | 4.57 | <.001 | L Posterior orbital gyrus | 40.19% |
|  |  |  |  |  |  |  |  | L Anterior orbital gyrus | 16.5% |
|  |  |  |  |  |  |  |  | L Medial orbital gyrus | 16.12% |
|  | 48 | -56 | -20 | .007 | 97 | 4.35 | <.001 | R Inferior Temporal Gyrus | 60.58% |
|  |  |  |  |  |  |  |  | R Fusiform Gyrus | 20.19% |
|  |  |  |  |  |  |  |  | R Cerebellum Crus 1 | 13.79% |
| Tool > Bar (Planning): Young | 28 | -90 | 6 | <.001 | 894 | 6.60 | <.001 | R Middle Occipital Gyrus | 59.42% |
|  |  |  |  |  |  |  |  | R Superior Occipital Gyrus | 11.84% |
|  | 22 | -98 | 0 |  |  | 6.16 | <.001 | R calcarine fissure | 38.64% |
|  |  |  |  |  |  |  |  | R Inferior Occipital Gyrus | 14.95% |
|  |  |  |  |  |  |  |  | R Middle Occipital Gyrus | 13.4% |
|  | Peak voxel coordinates in MNI space | | | *p_FWE corr_* | Cluster size | T value | *p_unc_* | AAL Labeling | Percentage of overlapping |
|  | X | Y | Z |  |  |  |  |  |  |
|  | 32 | -92 | -2 |  |  | 6.10 | <.001 | R Inferior Occipital Gyrus | 47.18% |
|  |  |  |  |  |  |  |  | R Middle Occipital Gyrus | 36.7% |
|  | -16 | -60 | 62 | <.001 | 3027 | 6.52 | <.001 | L Superior Parietal Gyrus | 52.23% |
|  |  |  |  |  |  |  |  | L Precuneus | 46.8% |
|  | -12 | -72 | 52 |  |  | 6.34 | <.001 | L Precuneus | 55.92% |
|  |  |  |  |  |  |  |  | L Superior Parietal Gyrus | 40.58% |
|  | -22 | -54 | 62 |  |  | 6.22 | <.001 | L Superior Parietal Gyrus | 79.61% |
|  |  |  |  |  |  |  |  | L Precuneus | 11.65% |
|  | -28 | -90 | 2 | <.001 | 2357 | 6.38 | <.001 | L Middle Occipital Gyrus | 79.61% |
|  |  |  |  |  |  |  |  | L Inferior Occipital Gyrus | 12.62% |
|  | -22 | -96 | -2 |  |  | 5.46 | <.001 | L Middle Occipital Gyrus | 57.67% |
|  |  |  |  |  |  |  |  | L Inferior Occipital Gyrus | 22.52% |
|  | -48 | -70 | -12 |  |  | 5.38 | <.001 | L Inferior Occipital Gyrus | 58.06% |
|  |  |  |  |  |  |  |  | L Inferior Temporal Gyrus | 14.17% |
|  | 32 | -50 | -22 | <.001 | 220 | 5.18 | <.001 | R Cerebellum 6 | 49.9% |
|  |  |  |  |  |  |  |  | R Fusiform Gyrus | 43.3% |
|  | 40 | -52 | -28 |  |  | 4.10 | <.001 | R Cerebellum Crus 1 | 40.97% |
|  |  |  |  |  |  |  |  | R Cerebellum 6 | 34.95% |
|  |  |  |  |  |  |  |  | R Fusiform Gyrus | 18.25% |
|  | 40 | -52 | -16 |  |  | 3.95 | <.001 | R Fusiform Gyrus | 63.3% |
|  |  |  |  |  |  |  |  | R Inferior Temporal Gyrus | 26.02% |
|  | 0 | -26 | 28 | <.001 | 192 | 4.85 | <.001 | R Middle Cingulate Gyrus | 18.45% |
|  |  |  |  |  |  |  |  | L Middle Cingulate Gyrus | 15.73% |
|  | -4 | -18 | 30 |  |  | 4.04 | <.001 | L Middle Cingulate Gyrus | 31.46% |
|  |  |  |  |  |  |  |  | R Middle Cingulate Gyrus | 11.07% |
|  | 4 | -34 | 26 |  |  | 3.59 | <.001 | R Middle Cingulate Gyrus | 16.7% |
|  |  |  |  |  |  |  |  | R Cingulate Post Gyrus | 12.04% |
|  |  |  |  |  |  |  |  | L Cingulate Post Gyrus | 11.65% |
|  | 18 | -64 | 64 | <.001 | 440 | 4.23 | <.001 | R Superior Parietal Gyrus | 74.17% |
|  |  |  |  |  |  |  |  | R Precuneus | 16.31% |
|  | 12 | -72 | 54 |  |  | 4.21 | <.001 | R Precuneus | 59.03% |
|  |  |  |  |  |  |  |  | R Superior Parietal Gyrus | 36.31% |
|  | 18 | -58 | 54 |  |  | 4.13 | <.001 | R Superior Parietal Gyrus | 56.12% |
|  |  |  |  |  |  |  |  | R Precuneus | 28.35% |
| Use > Trans (Planning): Elderly | 36 | -50 | -28 | <.001 | 105 | 4.78 | <.001 | R Cerebellum 6 | 59.22% |
|  |  |  |  |  |  |  |  | R Cerebellum Crus_1 | 22.14% |
|  |  |  |  |  |  |  |  | R Fusiform Gyrus | 17.86% |
|  | -24 | -8 | 48 | <.001 | 143 | 4.67 | <.001 | L Precentral Gyrus | 23.3 % |
|  |  |  |  |  |  |  |  | L Superior Frontal Gyrus | 17.86% |
|  | -22 | -12 | 58 |  |  | 3.93 | <.001 | L Precentral Gyrus | 36.31% |
|  |  |  |  |  |  |  |  | L Superior Frontal Gyrus | 21.94% |
|  | -28 | -94 | -8 | <.001 | 170 | 4.13 | <.001 | L Inferior Occipital Gyrus | 40.78% |
|  |  |  |  |  |  |  |  | L Middle Occipital Gyrus | 26.41% |
|  |  |  |  |  |  |  |  | L Lingual Gyrus | 10.29% |
|  | -20 | -100 | -2 |  |  | 3.35 | <.001 | L Middle Occipital Gyrus | 46.8% |
|  |  |  |  |  |  |  |  | L Inferior Occipital Gyrus | 15.15% |
|  |  |  |  |  |  |  |  | L Calcarine Fissure | 11.07% |
| Use > Trans (Planning): Young | No significant activation on cluster-level correction | | | | | | | | |
| Tool > Bar (Execution): Elderly | 0 | -4 | 58 | .037 | 139 | 4.11 | <.001 | L Supplementary Motor Area | 56.12% |
|  | Peak voxel coordinates in MNI space | | | *p_FWE corr_* | Cluster size | T value | *p_unc_* | AAL Labeling | Percentage of overlapping |
|  | X | Y | Z |  |  |  |  |  |  |
|  |  |  |  |  |  |  |  | R Supplementary Motor Area | 41.55% |
|  | -4 | 4 | 50 |  |  | 3.83 | <.001 | L Supplementary Motor Area | 68.74% |
|  |  |  |  |  |  |  |  | L Middle Cingulate Gyrus | 14.95% |
|  |  |  |  |  |  |  |  | R Supplementary Motor Area | 12.23% |
| Tool > Bar (Execution): Young | -28 | -96 | -6 | <.001 | 17736 | 9.40 | <.001 | L Middle Occipital Gyrus | 36.12% |
|  |  |  |  |  |  |  |  | L Inferior Occipital Gyrus | 31.07% |
|  | -36 | -92 | -6 |  |  | 9.20 | <.001 | L Middle Occipital Gyrus | 34.37% |
|  |  |  |  |  |  |  |  | L Inferior Occipital Gyrus | 22.72% |
|  | -18 | 2 | 58 |  |  | 7.74 | <.001 | L Superior Frontal Gyrus | 57.86% |
|  |  |  |  |  |  |  |  | L Supplementary Motor Area | 18.45% |
|  | 26 | -94 | -6 | <.001 | 816 | 7.61 | <.001 | R Inferior Occipital Gyrus | 44.27% |
|  |  |  |  |  |  |  |  | R Lingual Gyrus | 25.63% |
|  |  |  |  |  |  |  |  | R Calcarine Fissure | 16.7% |
|  | 34 | -92 | -6 |  |  | 6.68 | <.001 | R Inferior Occipital Gyrus | 65.63% |
|  |  |  |  |  |  |  |  | R Middle Occipital Gyrus | 15.53% |
|  | 22 | -100 | 6 |  |  | 5.85 | <.001 | R Calcarine Fissure | 21.36% |
|  |  |  |  |  |  |  |  | R Superior Occipital Gyrus | 16.31% |
|  |  |  |  |  |  |  |  | R Cuneus | 15.34% |
|  |  |  |  |  |  |  |  | R Middle Occipital Gyrus | 14.37% |
|  | -46 | 36 | 24 | <.001 | 506 | 6.30 | <.001 | L Middle Frontal Gyrus | 41.94% |
|  |  |  |  |  |  |  |  | L Inferior Frontal Gyrus, triangular part | 41.55% |
|  | -52 | 38 | 6 |  |  | 5.18 | <.001 | L Inferior Frontal Gyrus, triangular part | 76.12% |
|  | -48 | 44 | -2 |  |  | 4.68 | <.001 | L Inferior Frontal Gyrus, triangular part | 36.89% |
|  |  |  |  |  |  |  |  | L Middle Frontal Gyrus | 22.14% |
|  |  |  |  |  |  |  |  | L Inferior Frontal Orb | 22.14% |
|  | 56 | -18 | 54 | <.001 | 414 | 5.45 | <.001 | R Postcentral Gyrus | 44.47% |
|  |  |  |  |  |  |  |  | R Precentral Gyrus | 13.01% |
|  | 50 | -20 | 48 |  |  | 5.16 | <.001 | R Postcentral Gyrus | 72.62% |
|  |  |  |  |  |  |  |  | R Precentral Gyrus | 25.44% |
|  | 54 | -26 | 58 |  |  | 4.96 | <.001 | R Postcentral Gyrus | 45.05% |
|  | 24 | 10 | -4 | .002 | 246 | 5.02 | <.001 | R Putamen | 71.84% |
|  |  |  |  |  |  |  |  | R Pallidum | 10.29% |
|  | 8 | 8 | 4 |  |  | 4.10 | <.001 | R Caudate | 30.87% |
|  | 12 | 2 | 10 |  |  | 4.01 | <.001 | R Caudate | 29.9% |
|  | 56 | 16 | 20 | .015 | 171 | 4.74 | <.001 | R Inferior Frontal Gyrus, opercular part | 61.75% |
|  |  |  |  |  |  |  |  | R Inferior Frontal Gyrus, triangular part | 32.82% |
|  | 56 | 18 | 12 |  |  | 4.36 | <.001 | R Inferior Frontal Gyrus, opercular part | 59.61% |
|  |  |  |  |  |  |  |  | R Inferior Frontal Gyrus, triangular part | 39.42% |
|  | 60 | 10 | 32 |  |  | 4.19 | <.001 | R Precentral Gyrus | 50.68% |
|  |  |  |  |  |  |  |  | R Inferior Frontal Gyrus, opercular part | 27.57% |
|  | 36 | 22 | 0 | .001 | 282 | 4.72 | <.001 | R Insula | 59.81% |
|  |  |  |  |  |  |  |  | R Inferior Frontal Gyrus, triangular part | 10.87% |
|  | 52 | 16 | -10 |  |  | 4.22 | <.001 | R Superior Temporal Gyrus | 50.68% |
|  |  |  |  |  |  |  |  | R Inferior Frontal Gyrus, pars orbitalis | 15.53% |
|  | Peak voxel coordinates in MNI space | |  | *p_FWE corr_* | Cluster size | T value | *p_unc_* | AAL Labeling | Percentage of overlapping |
|  | X | Y | Z |  |  |  |  |  |  |
|  |  |  |  |  |  |  |  | R Insula | 14.95% |
|  | 42 | 18 | -14 |  |  | 4.21 | <.001 | R Insula | 37.67% |
|  |  |  |  |  |  |  |  | R Superior Temporal Gyrus | 21.55% |
|  |  |  |  |  |  |  |  | R Posterior orbital Gyrus | 19.81% |
|  |  |  |  |  |  |  |  | R Inferior Frontal Gyrus, pars orbitalis | 11.65% |
| Use > Trans (Execution): Elderly | No significant activation on cluster-level correction | | | | | | | | |
| Use > Trans (Execution): Young | -20 | -4 | 58 | <.001 | 513 | 5.12 | <.001 | L Superior Frontal Gyrus | 50.29% |
|  |  |  |  |  |  |  |  | L Supplementary Motor Area | 10.87% |
|  | -22 | 0 | 70 |  |  | 4.72 | <.001 | L Superior Frontal Gyrus | 71.46% |
|  | -32 | -14 | 60 |  |  | 4.58 | <.001 | L Precentral Gyrus | 94.17% |
|  | -44 | -86 | -4 | <.001 | 1363 | 5.05 | <.001 | L Middle Occipital Gyrus | 31.26% |
|  |  |  |  |  |  |  |  | L Inferior Occipital Gyrus | 18.45% |
|  | -38 | -90 | -12 |  |  | 4.70 | <.001 | L Inferior Occipital Gyrus | 21.94% |
|  | -40 | -86 | 18 |  |  | 4.67 | <.001 | L Middle Occipital Gyrus | 64.08% |
|  | -48 | 22 | 48 | .036 | 140 | 4.20 | <.001 | L Postcentral Gyrus | 77.28% |
|  |  |  |  |  |  |  |  | L Inferior Parietal Gyrus | 20.39% |
|  | -42 | -28 | 54 |  |  | 3.90 | <.001 | L Postcentral Gyrus | 86.6% |
|  |  |  |  |  |  |  |  | L Precentral Gyrus | 10.68% |
|  | -38 | -34 | 60 |  |  | 3.72 | <.001 | L Postcentral Gyrus | 86.41% |
|  |  |  |  |  |  |  |  | L Precentral Gyrus | 11.26% |
| Elderly > Young: Planning | -34 | 4 | 30 | <.001 | 1525 | 5.91 | <.001 | L Precentral Gyrus | 25.24% |
|  |  |  |  |  |  |  |  | L Inferior Frontal Gyrus, triangular part | 17.67% |
|  | -42 | 14 | 30 |  |  | 4.72 | <.001 | L Inferior Frontal Gyrus, triangular part | 33.79% |
|  |  |  |  |  |  |  |  | L Inferior Frontal Gyrus, opercular part | 29.51% |
|  |  |  |  |  |  |  |  | L Middle Frontal Gyrus | 21.17% |
|  |  |  |  |  |  |  |  | L Precentral Gyrus | 14.95% |
|  | -36 | 2 | 64 |  |  | 4.43 | <.001 | L Precentral Gyrus | 30.29% |
|  |  |  |  |  |  |  |  | L Middle Frontal Gyrus | 19.03% |
|  | 44 | 0 | 60 | <.001 | 2145 | 5.68 | <.001 | R Middle Frontal Gyrus | 51.65% |
|  | 40 | 10 | 34 |  |  | 5.21 | <.001 | R Inferior Frontal Gyrus, opercular part | 46.99% |
|  |  |  |  |  |  |  |  | R Middle Frontal Gyrus | 27.96% |
|  |  |  |  |  |  |  |  | R Precentral Gyrus | 20.19% |
|  | 48 | 2 | 52 |  |  | 4.72 | <.001 | R Middle Frontal Gyrus | 43.5% |
|  |  |  |  |  |  |  |  | R Precentral Gyrus | 39.42% |
|  | 38 | -84 | -4 | <.001 | 1907 | 5.05 | <.001 | R Inferior Occipital Gyrus | 64.27% |
|  |  |  |  |  |  |  |  | R Middle Occipital Gyrus | 25.44% |
|  | 28 | -88 | 2 |  |  | 4.61 | <.001 | R Middle Occipital Gyrus | 41.36% |
|  |  |  |  |  |  |  |  | R Occipital Inferior Gyrus | 15.73% |
|  | 26 | -94 | 10 |  |  | 4.60 | <.001 | R Middle Occipital Gyrus | 45.83% |
|  |  |  |  |  |  |  |  | R Occipital Superior Gyrus | 28.35% |
|  |  |  |  |  |  |  |  | R Cuneus | 12.23% |
|  | 56 | -30 | -12 | .011 | 309 | 4.61 | <.001 | R Middle Temporal Gyrus | 65.44% |
|  |  |  |  |  |  |  |  | R Inferior Temporal Gyrus | 19.42% |
|  | 62 | -44 | -12 |  |  | 3.71 | <.001 | R Inferior Temporal Gyrus | 56.7% |
|  |  |  |  |  |  |  |  | R Middle Temporal Gyrus | 42.52% |
|  | Peak voxel coordinates in MNI space | | | *p_FWE corr_* | Cluster size | T value | *p_unc_* | AAL Labeling | Percentage of overlapping |
|  | X | Y | Z |  |  |  |  |  |  |
|  | 50 | -22 | -14 |  |  | 3.15 | <.001 | R Middle Temporal Gyrus | 34.17% |
|  |  |  |  |  |  |  |  | R Inferior Temporal Gyrus | 17.28% |
|  | -38 | -50 | 40 | <.001 | 983 | 4.59 | <.001 | L Inferior Parietal Gyrus | 69.9.% |
|  |  |  |  |  |  |  |  |  |  |
|  |  |  |  |  |  |  |  |  |  |
|  |  |  |  |  |  |  |  | L Angular Gyrus | 20% |
|  | -38 | -48 | 48 |  |  | 4.30 | <.001 | L Inferior Parietal Gyrus | 96.31% |
|  | -42 | -42 | 42 |  |  | 4.24 | <.001 | L Inferior Parietal Gyrus | 84.85% |
|  | -32 | -92 | 6 | .024 | 258 | 4.45 | <.001 | L Middle Occipital Gyrus | 81.94% |
|  | -38 | -84 | 4 |  |  | 3.68 | <.001 | L Middle Occipital Gyrus | 87.96% |
|  | -46 | -80 | -6 |  |  | 3.66 | <.001 | L Inferior Occipital Gyrus | 39.42% |
|  |  |  |  |  |  |  |  | L Middle Occipital Gyrus | 23.69% |
|  | 34 | -56 | 48 | <.001 | 631 | 4.38 | <.001 | R Angular Gyrus | 39.42% |
|  |  |  |  |  |  |  |  | R Inferior Parietal Gyrus | 37.86% |
|  |  |  |  |  |  |  |  | R Superior Parietal Gyrus | 17.48% |
|  | 36 | -54 | 40 |  |  | 4.12 | <.001 | R Angular Gyrus | 41.17% |
|  |  |  |  |  |  |  |  | R Inferior Parietal Gyrus | 32.04% |
|  | 32 | -72 | 32 |  |  | 4.05 | <.001 | R Middle Occipital Gyrus | 65.83% |
|  |  |  |  |  |  |  |  | R Superior Occipital Gyrus | 28.16% |
|  | 22 | -60 | 8 | .002 | 445 | 3.96 | <.001 | R Calcarine Fissure | 74.95% |
|  |  |  |  |  |  |  |  | R Lingual Gyrus | 18.06% |
|  | -28 | -70 | -14 |  |  | 3.67 | <.001 | L Fusiform Gyrus | 55.34% |
|  |  |  |  |  |  |  |  | L lobule 6 of cerebellar hemisphere | 25.44% |
|  |  |  |  |  |  |  |  | L Lingual Gyrus | 11.07% |
|  | -10 | -68 | 6 |  |  | 3.66 | <.001 | L Calcarine Fissure | 58.06% |
|  |  |  |  |  |  |  |  | L Lingual Gyrus | 41.94% |
|  |  |  |  |  |  |  |  |  |  |
| Young > Elderly: Planning | No significant activation on cluster-level correction | | | | | | | | |
| Elderly > Young: Execution | 20 | -6 | 78 | <.001 | 1019 | 11.80 | <.001 | R Superior Frontal Gyrus | 33.59% |
|  | 26 | -34 | 78 |  |  | 11.15 | <.001 | R Postcentral Gyrus | 46.8% |
|  |  |  |  |  |  |  |  | R Precentral Gyrus | 11.46% |
|  | 34 | -26 | 74 |  |  | 11.08 | <.001 | R Precentral Gyrus | 38.25% |
|  |  |  |  |  |  |  |  | R Postcentral Gyrus | 12.82% |
|  | 18 | -34 | 24 | <.001 | 918 | 11.06 | <.001 | NaN |  |
|  | 10 | -20 | 26 |  |  | 7.39 | <.001 | R Middle Cingulate Gyrus | 15.34% |
|  | 32 | -48 | 12 |  |  | 5.70 | <.001 | NaN |  |
|  | -56 | -64 | -14 | <.001 | 4033 | 10.86 | <.001 | L Inferior Temporal Gyrus | 44.27% |
|  |  |  |  |  |  |  |  | L Inferior Occipital Gyrus | 17.48% |
|  | -54 | -52 | -22 |  |  | 9.07 | <.001 | L Inferior Temporal Gyrus | 78.83% |
|  |  |  |  |  |  |  |  | L Crus 1 of cerebellar hemisphere | 10.29% |
|  | -64 | -36 | -14 |  |  | 8.95 | <.001 | L Middle Temporal Gyrus | 60.19% |
|  |  |  |  |  |  |  |  | L Inferior Temporal Gyrus | 32.23% |
|  | -28 | 22 | -24 |  | 685 | 10.74 | <.001 | L Posterior orbital gyrus | 50.29% |
|  |  |  |  |  |  |  |  | L Superior Temporal Gyrus | 30.87% |
|  | -34 | 30 | -20 |  |  | 10.14 | <.001 | L Posterior Orbital Gyrus | 46.12% |
|  | 0 | 30 | -22 |  |  | 8.31 | <.001 | L Gyrus Rectus | 47.77% |
|  |  |  |  |  |  |  |  | R Gyrus Rectus | 29.32% |
|  | Peak voxel coordinates in MNI space | | | *p_FWE corr_* | Cluster size | T value | *p_unc_* | AAL Labeling | Percentage of overlapping |
|  | X | Y | Z |  |  |  |  |  |  |
|  | 36 | 44 | -16 | .001 | 542 | 9.57 | <.001 | R Anterior orbital gyrus | 43.5% |
|  |  |  |  |  |  |  |  | R Middle Frontal Gyrus | 16.12% |
|  | 44 | 46 | -14 |  |  | 7.94 | <.001 | R Inferior Frontal Gyrus, pars orbitalis | 23.11% |
|  |  |  |  |  |  |  |  | R Anterior Orbital Gyrus | 20.97% |
|  |  |  |  |  |  |  |  | R Middle Frontal Gyrus | 18.25% |
|  |  |  |  |  |  |  |  | R Lateral Orbital Gyrus | 16.31% |
|  | 36 | 32 | -18 |  |  | 6.39 | <.001 | R Posterior Orbital Gyrus | 49.71% |
|  |  |  |  |  |  |  |  | R Inferior Frontal Orb Gyrus | 15.15% |
|  |  |  |  |  |  |  |  | R Anterior Orbital Gyrus | 14.37% |
|  | -42 | -74 | -38 | .002 | 447 | 9.48 | <.001 | L Crus 2 of cerebellar hemisphere | 48.93% |
|  |  |  |  |  |  |  |  | L Crus 1 of cerebellar hemisphere | 38.45% |
|  | -24 | -86 | -36 |  |  | 6.49 | <.001 | L Crus 2 of cerebellar hemisphere | 56.89% |
|  |  |  |  |  |  |  |  | L Crus 1 of cerebellar hemisphere | 19.22% |
|  | -12 | -84 | -38 |  |  | 6.19 | <.001 | L Crus 2 of cerebellar hemisphere | 71.26% |
|  | 20 | -96 | -14 | <.001 | 1935 | 9.36 | <.001 | R Lingual Gyrus | 45.83% |
|  |  |  |  |  |  |  |  | R Inferior Occipital Gyrus | 10.29% |
|  | 42 | -84 | -12 |  |  | 8.65 | <.001 | R Inferior Occipital Gyrus | 66.21% |
|  | 12 | -98 | -2 |  |  | 5.82 | <.001 | R Calcarine Fissure | 43.88% |
|  |  |  |  |  |  |  |  | R Lingual Gyrus | 10.68% |
|  | 68 | -32 | -12 | .002 | 425 | 8.10 | <.001 | R Middle Temporal Gyrus | 67.18% |
|  |  |  |  |  |  |  |  | R Inferior Temporal Gyrus | 16.5% |
|  | 28 | -24 | -26 |  |  | 7.60 | <.001 | R Parahippocampal Gyrus | 42.72% |
|  |  |  |  |  |  |  |  | R Fusiform Gyrus | 19.81% |
|  |  |  |  |  |  |  |  | R Lobule 4,5 of cerebellar hemisphere | 15.34% |
|  | 54 | -30 | -20 |  |  | 7.52 | <.001 | R Inferior Temporal Gyrus | 70.49% |
|  |  |  |  |  |  |  |  | R Middle Temporal Gyrus | 13.98% |
|  | -14 | -58 | -28 | .006 | 349 | 6.44 | <.001 | L Lobule 6 of cerebellar hemisphere | 36.7% |
|  | -30 | -50 | -38 |  |  | 5.29 | <.001 | L Lobule 6 of cerebellar hemisphere | 25.63% |
|  |  |  |  |  |  |  |  | L Lobule 8 of cerebellar hemisphere | 20.19% |
|  |  |  |  |  |  |  |  | L Crus 1 of cerebellar hemisphere | 14.17% |
|  | -22 | -66 | -34 |  |  | 3.40 | <.001 | L Crus 1 of cerebellar hemisphere | 38.83% |
|  |  |  |  |  |  |  |  | L Lobule 6 of cerebellar hemisphere | 23.3% |
|  |  |  |  |  |  |  |  | L Lobule 8 of cerebellar hemisphere | 13.2% |
|  | 14 | -60 | -28 | <.001 | 639 | 5.37 | <.001 | R Lobule 6 of cerebellar hemisphere | 40.78% |
|  | 8 | -84 | -34 |  |  | 5.35 | <.001 | R Crus 2 of cerebellar hemisphere | 72.82% |
|  | 38 | -50 | -40 |  |  | 5.34 | <.001 | R Crus 1 of cerebellar hemisphere | 34.37% |
|  |  |  |  |  |  |  |  | R Crus 2 of cerebellar hemisphere | 16.5% |
|  |  |  |  |  |  |  |  | R Lobule 8 of cerebellar hemisphere | 15.92% |
|  | Peak voxel coordinates in MNI space | | | *p_FWE corr_* | Cluster size | T value | *p_unc_* | AAL Labeling | Percentage of overlapping |
|  | X | Y | Z |  |  |  |  |  |  |
| Young > Elderly: Execution | -10 | 20 | 6 | <.001 | 68838 | 18.56 | <.001 | L Caudate | 53.59% |
|  | 14 | -10 | 18 |  |  | 17.38 | <.001 | R Caudate | 21.17% |
|  |  |  |  |  |  |  |  | R Thalamus, ventral lateral nucleus | 20% |
|  | 10 | 22 | 4 |  |  | 17.23 | <.001 | R Caudate | 43.11% |
|  |  |  |  |  |  |  |  |  |  |

## Appendix B: Laterality indices for all conditions during the planning phase

##

Figure 1: Calculated laterality indices for all conditions during the planning phase. One-sample t-test results are indicated, p<.05* and p<.001**, respectively.

In Figure 1 calculated laterality indices for all conditions (Tool use, Tool transport, Bar use, Bar transport) during the planning phases are depicted for each group. Positive values indicate left-lateralization, whereas negative values indicate right-lateralization. One-sample t-test with Bonferroni correction was applied, in order to test significant differences of LI (laterality index) values from 0 (indicating symmetrical activation).

## Appendix C: Laterality Indices for all conditions during the execution phase

Figure 2: Calculated laterality indices for all conditions during the execution phase. One-sample t-test results are indicated, p<.05* and p<.001**, respectively.

In Figure 2 calculated laterality indices for all conditions (Tool use, Tool transport, Bar use, Bar transport) during the execution phases are depicted for each group. Positive values indicate left-lateralization, whereas negative values indicate right-lateralization. One-sample t-test with Bonferroni correction was applied, in order to test significant differences of LI (laterality index) values from 0 (indicating symmetrical activation).

## Appendix D: DMN and L-FPN Network

|  | Brain Regions | Centroid coordinates in MNI space | | |
| --- | --- | --- | --- | --- |
| Left lateral frontoparietal network (L-FPN) | Pre-supplementary motor area/ dorsal anterior cingulate cortex | -2 | 24 | 38 |
|  | left inferior parietal lobe/ precuneus | -36 | -48 | 38 |
|  | left anterior insula | -32 | 18 | -4 |
|  | Left dorsolateral prefrontal cortex | -50 | 32 | 26 |
| Default mode network (DMN) | Left temporal_1 | -56 | -4 | -20 |
|  | Left temporal_2 | -58 | -32 | -2 |
|  | Left parietal_1 | -58 | -50 | 12 |
|  | Left parietal_2 | -48 | -64 | 36 |
|  | Left prefrontal Cortex_1 | -34 | 22 | -10 |
|  | Left prefrontal Cortex_2 | -46 | 34 | -4 |
|  | Left prefrontal Cortex_3 | -6 | 46 | 0 |
|  | Left prefrontal Cortex_4 | -24 | 60 | -2 |
|  | Left prefrontal Cortex_5 | -8 | 48 | 42 |
|  | Left prefrontal Cortex_6 | -40 | 14 | 48 |
|  | Left prefrontal Cortex_7 | -26 | 20 | 52 |
|  | Left precuneus posterior cingulate cortex_1 | -12 | -56 | 12 |
|  | Left precuneus posterior cingulate cortex_2 | -6 | -52 | 34 |
|  | Right parietal_1 | 54 | -50 | 30 |
|  | Right temporal_1 | 62 | -24 | -18 |
|  | Right temporal_2 | 50 | 6 | -18 |
|  | Right temporal_3 | 58 | -26 | -2 |
|  | Right ventral prefrontal cortex_1 | 36 | 26 | -16 |
|  | Right ventral prefrontal cortex_2 | 50 | 28 | 0 |
|  | Right dorsal and medial prefrontal cortex_1 | 6 | 48 | 0 |
|  | Right dorsal and medial prefrontal cortex_2 | 12 | 50 | 40 |
|  | Right dorsal and medial prefrontal cortex_3 | 26 | 24 | 50 |
|  | Right precuneus posterior cingulate cortex_1 | 12 | -54 | 14 |
|  | Right precuneus posterior cingulate cortex_2 | 6 | -52 | 30 |

Table 1: Centroid coordinates were extracted for L-FPN Dajani et al. (2020), and DMN, Schaefer et al. (2018); 6mm spheres surrounded these centroid coordinates.


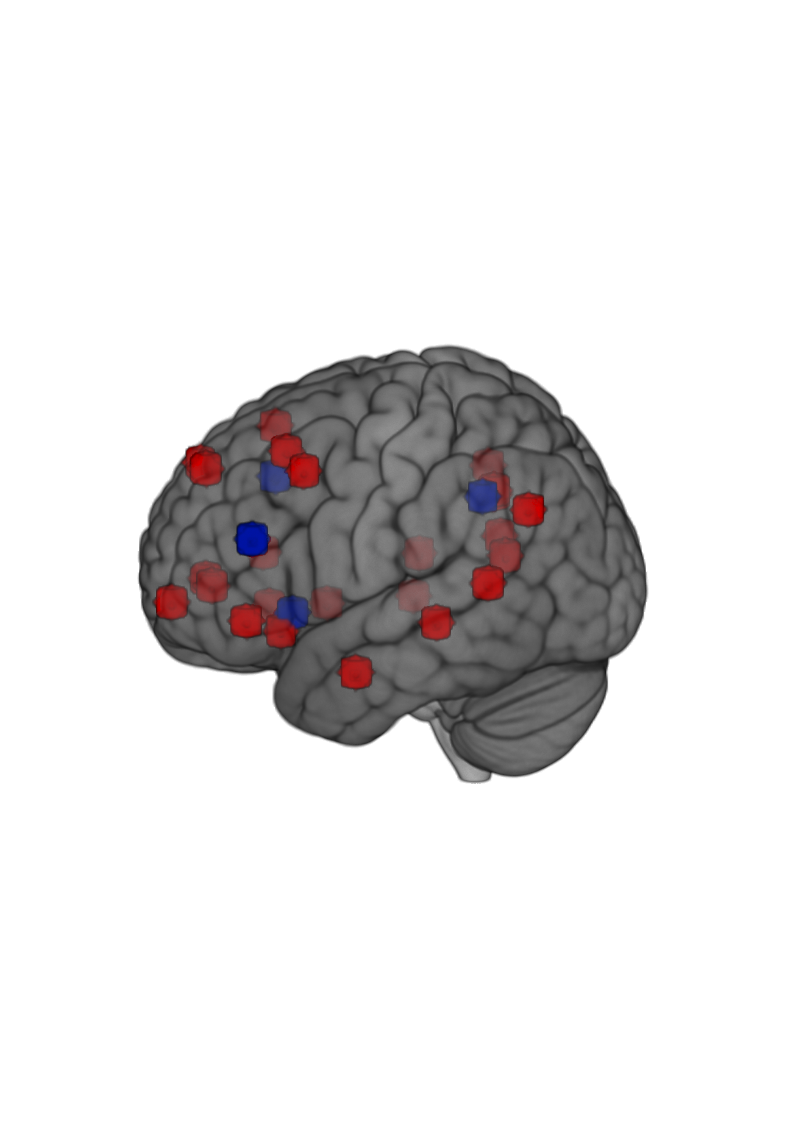

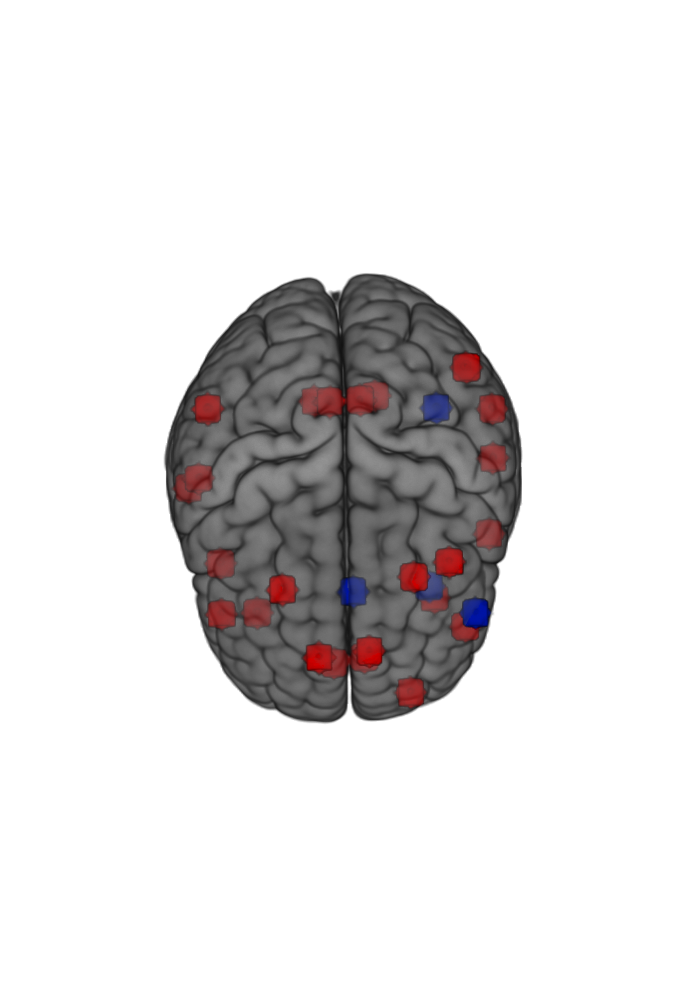


Figure 3: Depiction of the created masks; blue (regions for L-FPN according to Dajani et al. (2020)) and red (regions for DMN, according to Schaefer et al., (2018)).

For calculating the relationship between the default mode network during planning and the left lateral frontoparietal network during execution, MNI coordinates were extracted from previous literature (Dajani et al. (2020) and Schaefer et al. (2018)). The created masks were then used in order to extract the according beta-weights from the group-level, general linear model for the contrasts tool use and bar use for both groups.
